# LOX-1: Implications in atherosclerosis and myocardial ischemia

**DOI:** 10.17179/excli2021-4532

**Published:** 2022-01-13

**Authors:** Tanya Sharma, Francesco Romeo, Jawahar L. Mehta

**Affiliations:** 1 Division of Cardiology, Central Arkansas Veterans Healthcare System and the University of Arkansas for Medical Sciences, Little Rock, AR 72205, USA; 2 Department of Cardiology, International University Unicamillus, Rome, Italy

**Keywords:** atherosclerosis, myocardial ischemia, oxidized-LDL, LOX-1

## Abstract

Understanding the pathophysiology of atherosclerosis is fundamental to the practice of cardiovascular medicine. Atherosclerosis is a multi-step cascade of accumulation of lipids and downstream changes that lead to a fibro-fatty plaque formation in the arterial intima. Multiple biochemical stimuli, cellular receptors and intra-cellular signals are implicated in this complex mechanism. Lectin-type oxidized LDL receptor-1 or LOX-1 is a type II membrane glycoprotein receptor which has emerged as an important effector of atherosclerosis. Hence, LOX-1 modification and its clinical consequences are of much interest in recent times.

## Introduction

Atherosclerosis is the result of a complex cascade. It occurs in an oxidative environment and the first step of the cascade is the modification of low-density lipoprotein (LDL) to oxidized LDL (ox-LDL) which binds to a variety of scavenger receptors (SRs) on the endothelium and monocytes/macrophages. Formation of ox-LDL and its uptake into the intimal cells is a crucial step and has several downstream effects, including generation of reactive oxygen species (ROS), inhibition of the constitutive endothelial nitric oxide synthetase (eNOS) enzyme in the endothelium, chemotaxis and adhesion of monocytes to the endothelial cells, and disruption of fibrous cap of the atheroma leading to the formation of platelet-leukocytes rich clots in the narrowed atherosclerotic artery and resultant adverse clinical events (Goyal et al., 2012[7]; Roy Chowdhury et al., 2010[21]; Cominacini et al., 2001[4]). These processes are mediated by binding of ox-LDL to cell surface SRs. LOX-1 is one such SR which was described by Sawamura et al. in 1997. LOX-1 is prominently expressed on the endothelial cells. It is 50 kD transmembrane glycoprotein involved in binding, internalization, and proteolytic degradation of ox-LDL (Sawamura et al., 1997[22]). 

It was first studied on bovine aortic-endothelial cells and subsequently found to be abundantly expressed in human aortic intima and vascular-rich organs like placenta, lung, brain, and liver (Sawamura et al., 1997[22]). Its presence on coronary artery endothelial cells was described by our group in 1998 (Mehta and Li, 1998[18]). Other cells that express LOX-1 include macrophages, platelets, fibroblasts, and vascular smooth muscle cells (SMCs) that explain multi-level involvement of LOX-1 in atherosclerosis (Chen et al., 2001[3]; Kataoka et al., 2001[12]; Yoshida et al., 1998[25]).

## LOX-1 Genetics and Expression

LOX-1 belongs to the C-type lectin receptor family and its structure is noted to be highly conserved across mammalian species. In humans, it is coded by a single-copy gene, *OLR1 *(oxidized low-density lipoprotein receptor 1, HGNC:8133) located on the short arm of chromosome 12 (Aoyama et al., 1999[1]). Polymorphisms in the OLR1 gene have been described that are associated with susceptibility to atherosclerosis (Mango et al., 2011[17]). LOX-1 has an inducible expression. Ox-LDL, pro-inflammatory cytokines like angiotensin-II, tumor necrosis factor-α, advanced glycation end-products, and mechanical factors like shear stress can lead to upregulation of LOX-1 expression (Murase et al., 1998[20]; Kume et al., 1998[13]). The presence of genetic variants in nature is a valuable cue to future research in pharmacogenetics to prevent and treat atherosclerosis.

## Implications in Clinical Disease States

Although LOX-1 activation has been pathological implicated in a host of disease states, incusing development of atherosclerosis, myocardial ischemia, renal dysfunction resulting from ischemia and neuro-inflammatory diseases, for sake of simplicity, we will focus on atherosclerosis and myocardial ischemia.

### Atherosclerosis

**Figure 1:**
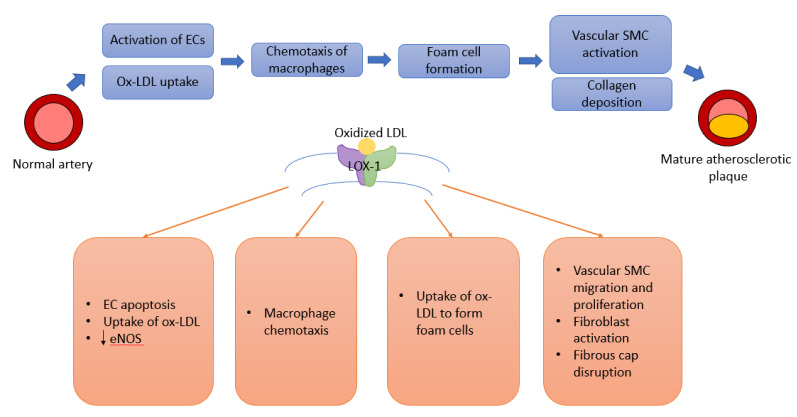
Pathway leading to atherosclerosis and the role of LOX-1. Abbreviations: EC: Endothelial cell; eNOS: endothelial nitric oxide synthase; LOX-1: Lectin-type oxidized LDL receptor-1; ox-LDL: Oxidized-Low density lipoprotein; SMC: Smooth muscle cell

Figure 1(Fig. 1) shows the atherosclerotic cascade leading to formation of a mature fibro-fatty plaque and the role played by LOX-1 at every stage of the pathway. Owing to its central position in the pathogenetic mechanism, LOX-1 is an attractive focus of manipulation to regulate atherosclerosis. In endothelial cells, LOX-1 is the primary receptor responsible for ox-LDL. Over several experiments in LDL receptor deficient mice, our group found that deletion of LOX-1 ameliorates oxLDL-mediated endothelial dysfunction and inhibits atherogenesis (Mehta et al., 2007[19]). Table 1(Tab. 1) (References in Table 1: Eto et al., 2006[6]; Hong et al., 2014[9]; Hu et al., 2008[10]; Li and Mehta, 2000[14]; Mehta et al., 2007[19]) summarizes this and a few important studies utilizing LOX-1 inhibition to demonstrate significant changes in atherosclerosis.

### Myocardial ischemia

**Figure 2:**
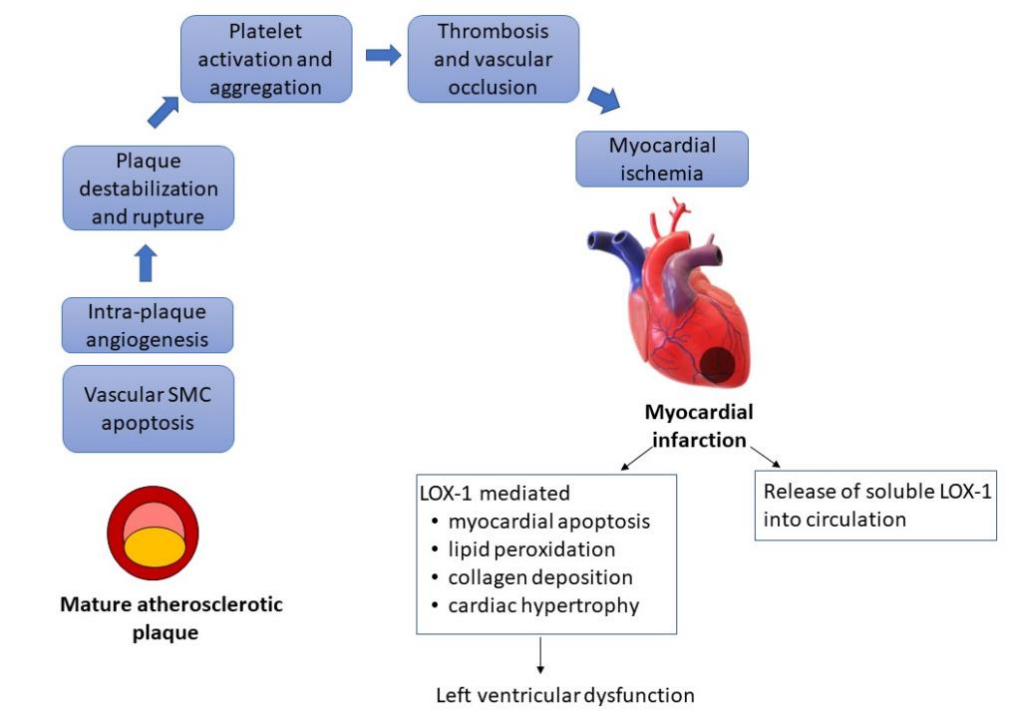
LOX-1 and the ischemic cascade

The formation of a mature atherosclerotic plaque is a prerequisite for acute ischemic events. However, not everyone with atherosclerosis experiences an ischemic event. It is the downstream clinical events that include destabilization and rupture of the plaque leading to platelet activation, thrombosis and vascular occlusion that leads to myocardial ischemia. The role of LOX-1 is implicated in this part of the ischemic cascade as well and the consequences of LOX-1 modification are delineated in Table 2(Tab. 2) and Figure 2(Fig. 2).

Angiogenesis occurs within the growing plaque and is presumed to be due to local ischemia and inflammation. The neo-capillaries are fragile and destabilize the plaque. Angiotensin-II is a cytokine which induces the expression of LOX-1 which in part contributes to intra-plaque angiogenesis. LOX-1 deletion leads to decreased angiogenesis (Wang et al., 2014[24]). Ox-LDL also leads to vascular smooth muscle cell apoptosis which leads to plaque destabilization and rupture, which is also mediated by LOX-1 and decreased in LOX-1 knock out mice (Ding et al., 2013[5]). Rupture of an unstable plaque exposes a lipid rich milieu which is a nidus for thrombosis. Platelets express LOX-1 and are activated by ox-LDL. LOX-1 inhibition *in vitro* leads to reduction of thrombus growth (Carnevale et al., 2014[2]) 

LOX-1 expression on atheroma-derived cells and its release into circulation after myocardial ischemia, make it a potential marker of acute ischemia which can be clinically utilized (Hayashida et al., 2005[8]). LOX-1 expression is upregulated on myocardial cell exposed to short period of ischemia followed by reperfusion in animal models. The extent of myocardial injury can also be modified by LOX-1 modification, which is summarized in Table 2(Tab. 2) (References in Table 2: Carnevale et al., 2014[2]; Ding et al., 2013[5]; Li et al., 2003[15]; Lu et al., 2012[16]; Wang et al., 2014[24]). 

## Clinical Implications in Cardiovascular Medicine

While results of *in vitro* studies have been clearly conclusive, efforts to utilize this knowledge clinically are still underway. Inhibition on LOX-1 using silencing messenger ribonucleic acid (mRNA) or monoclonal antibodies in human models is challenging. LOX-1 molecule is highly conserved across mammalian species as described earlier. Chimeric antibodies using chicken to produce antibodies against recombinant human LOX-1 receptor have shown some success at decreasing ox-LDL uptake in human cells (Iwamoto et al., 2009[11]). Synthetic inhibitors binding with the hydrophobic tunnel of LOX-1 are under investigation. Thakkar et al. developed a large chemical database of such molecules two of which were shown to lead to decreased expression of LOX-1 mRNA on human umbilical vein endothelial cells. While there is no evidence of cell toxicity of these molecules, pharmacokinetics and other practical details of the molecules are still being studied and could be a huge leap in the prevention of atherosclerosis if clinically viable (Thakkar et al., 2015[23]).

## Conclusion

While the focus in prevention of atherosclerosis-related diseases currently lays heavily on modifying hyperlipidemia as a risk factor, the emphasis on only reduction of native LDL is not sufficient. Understanding the pathophysiology of atherosclerosis and the central role LOX-1 is critical. LOX-1 inhibitors hold the potential to be the next big breakthrough in the field of cardiovascular medicine.

## Figures and Tables

**Table 1:**
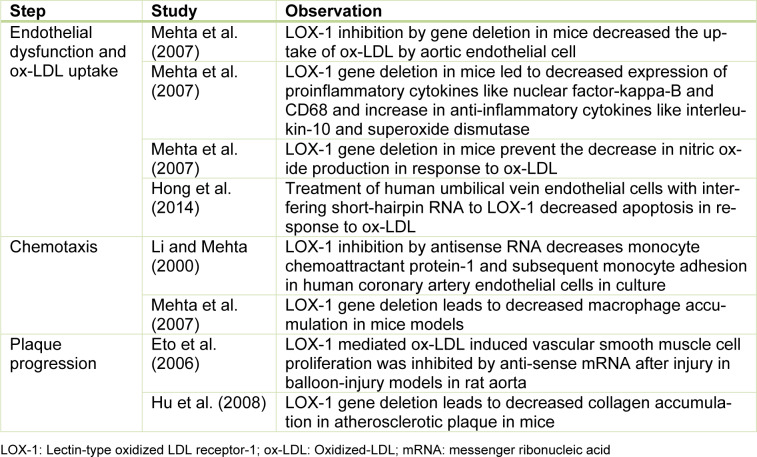
Important observations about the impact of LOX-1 inhibition on different steps of atherosclerotic plaque formation

**Table 2:**
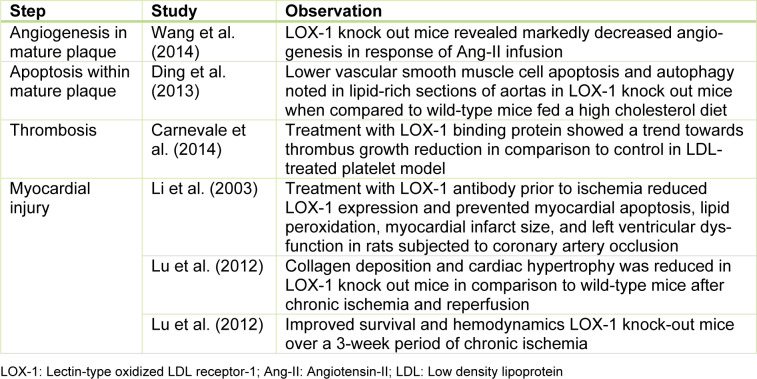
Effect of LOX-1 inhibition on the ischemic cascade leading to myocardial infarction
